# Clinical value of baseline ^18^F-FDG PET/CT in soft tissue sarcomas

**DOI:** 10.1186/s41824-021-00110-5

**Published:** 2021-09-03

**Authors:** Rafael Hernando Reyes Marlés, José Luis Navarro Fernández, José Pablo Puertas García-Sandoval, Fernando Santonja Medina, Laroussi Mohamed Salem, Laura Frutos Esteban, José Fulgencio Contreras Gutiérrez, María Isabel Castellón Sánchez, Guadalupe Ruiz Merino, María Antonia Claver Valderas

**Affiliations:** 1 Nuclear Medicine Division (DIMEC), Hospitales Universitarios San Roque, Las Palmas de Gran Canaria, Las Palmas Spain; 2 Nuclear Medicine Department, Hospital Clínico Universitario Virgen de la Arrixaca, El Palmar, Murcia Spain; 3 Orthopedics and Traumatology Department, Hospital Clínico Universitario Virgen de la Arrixaca, El Palmar, Murcia Spain; 4 Data Analytics Department, Instituto Murciano de Investigación Biosanitaria (IMIB) Virgen de la Arrixaca, El Palmar, Murcia Spain

**Keywords:** FDG, PET/CT, SUV, MTV, TLG, FNCLCC, Soft tissue sarcomas

## Abstract

**Background:**

The use of ^18^F-FDG Positron emission tomography/Computed tomography (PET/CT) in the initial staging of many cancers is clearly established. Most soft tissue sarcoma (STS) has a high affinity for ^18^F-FDG, which is why ^18^F-FDG PET/CT has been proposed as a non-invasive method, useful in diagnosis and follow-up. The standardized uptake value values (SUV), the volume-based metabolic parameters MTV (metabolic tumor volume), and TLG (total lesion glycolysis) determine tumor viability and provide its total volume and the total activity of metabolically active tumor cells. The histological grade is the most important predictor of metastases and mortality associated with STS, and a significant relationship between the metabolic parameters of ^18^F-FDG PET/CT and the histological grade has been described.

**Methods:**

A retrospective study was conducted on STS patients, who had histological grade according to the FNCLCC (Fédération Nationale des Centres de Lutte Contre Le Cancer) criteria, as well as a baseline PET/CT. SUV (SUV_max_, SUV_mean_, and SUV_peak_), MTV, and TLG were quantified. A T-student test was performed to establish the relationship between the metabolic biomarkers and the histological grade. Their usefulness as predictors of the histological grade was verified using receiver operator characteristic (ROC) curves. A survival function study was performed using the Kaplan–Meier method. To assess the prognostic utility of the metabolic biomarkers we use the Log-Rank method.

**Results:**

The SUV values were useful to discriminate high-grade STS. We found a significant relationship between the histological grade and the SUV values. SUV_max_, SUV_peak_, MTV, and TLG were predictors of overall survival (OS). There were no significant differences in the OS for the SUV_mean_, or in the disease-free survival (DFS) for SUV_max_, SUV_mean_, SUV_peak_, MTV, and TLG.

**Conclusions:**

The SUV_max_, SUV_mean_, and SUV_peak_ values correlate with the HG and are useful to discriminate high-grade from low-grade STS. Patients with high SUV_max_, SUV_peak_, MTV, and TLG have a significantly lower OS.

## Introduction

Soft tissue sarcomas (STS) are a group of rare tumors derived from mesenchymal tissues, whose cells develop in the circulatory and lymphatic systems, as well as in structural and connective tissues. Tumors that derive from peripheral nerves by convention are also classified as sarcomas, despite their embryological origin in the neural crest. Their great variety is due to the tissues in which they originate, with diverse clinical and biological behavior (Beckingsale and Shaw 2017). In Spain, the incidence rate of sarcomas is 18 to 20 cases per million in children, of which 6.5% are STS (Sarcomas en la Infancia 2020), with 2.000 total cases/year (Acciones and para que el Gobierno actúe sobre el Sarcoma (plan 2019–2020)). In Europe the estimated incidence is 5 new cases/100.000 inhabitants/year, corresponding to 1% of neoplasms in adults (Guerra Merino 2017). In the United States (U.S.), 13,130 new STS were diagnosed (7240 males and 5510 females) in 2020 (Key Statistics for Soft Tissue Sarcomas 2019). Both sexes are equally affected; the incidence is higher in females in the 45–49 age group, due to gynecological tumors (Beckingsale and Shaw 2017; López-Pousa et al. 2016). Liposarcoma and leiomyosarcoma are the most frequent STS (Francis et al. 1996; Stiller et al. 2013; Corey et al. 2014). Limbs are the most frequent location. However, the anatomic location varies according to the histological classification of the tumor (Bray et al. 2017; Brennan et al. 2014). In the United States, the most favorable survival rates are for dermatofibrosarcoma (92% 5-year survival), with a worse prognosis for undifferentiated chondrosarcoma (5-year survival 19%) (Corey et al. 2014). In Europe, the highest survival rate is for cutaneous STS (90%), while those of the mediastinum and heart have survival rates of less than 15% (Stiller et al. 2013). According to the National Cancer Institute (NCI), survival is determined by stage. The 5-year relative survival rates are 81% in stage 1 (localized), 58% in stage 2 (lymphatic spread), 16% in stage 3 (distant metastases), and 55% in unstaged sarcomas (National Cancer Society (NCS) 2020).

### Classification and grading

Pathological diagnosis, according to morphology, immunohistochemistry, and molecular analysis, provides data for the prognosis, quality of surgical resection, and treatment response (Villalobos León 2013). STS are classified according to the criteria of the World Health Organization (WHO), embodying data of molecular cytogenetics and pathogenesis (Fletcher et al. 2013; Vilanova 2017). The histological type doesn’t provide enough prognostic information. The histological grade is the most important predictor of metastases and mortality associated with STS (Fletcher et al. 2002). Trojani et al. (1984), established the pathological criteria correlated with the advent of metastases and survival and set up a grading system based on differentiation, mitotic count, and tumor necrosis, published by the FNCLCC (Fédération Nationale des Centres de Lutte Contre Le Cancer). FNCLCC grading system assigns a score according to the histological criteria, the sum of which determines the histological grade (grade 1, 2, and 3) (Coindre et al. 2001). The Union for International Cancer Control (UICC) (Brierley et al. 2017) summarizes the grading of STS in a two-grade system (FNCLCC grades 2 and 3 are classified as high-grade, and grade 1 as a low-grade).

### The role of ^18^F-FDG PET/CT in soft tissue sarcoma

Nuclear medicine provides metabolic information as an indirect measure of cellular function, based on the interaction of tissues with radioactive elements (Isabel and José 2020). The hybrid imaging, and mass production of 2-deoxy-2-[18F] fluoro-D-glucose (^18^F-FDG), improved the accuracy and diagnostic performance of molecular imaging, increasing oncology indications for Positron emission tomography/Computed tomography (PET/CT).

The ^18^F-FDG accumulation is mediated by the Glucose-transporter family (GLUT), located in the cell membranes, and regulates the uptake of glucose. The most important subtypes (GLUT 1 and GLUT 3) are overexpressed in tumors. Tumor cells need more glucose for proliferation than physiological cells because of the ineffective aerobic glycolysis (Warburg effect). The existence of this metabolic switch such reprogramming of cell energy metabolism must compensate for the lower efficiency of ATP production upregulating glucose transporters, which suggests that metabolic protein expression is associated with tumor aggressiveness and treatment response (Meyer et al. 2019). GLUT is also expressed in sarcomas (e.g., 50% of intrauterine leiomyosarcomas and 25% of extrauterine sarcomas by immunohistochemistry), and his positivity correlates closely with aggressive biological behavior, reflected by distant metastatic spread. In some cases, there was no detectable expression of GLUT in sarcoma, which suggests that another glucose transporter maintains glycolytic metabolism in these tumors, or that GLUT is expressed at specific stages of carcinogenesis. Some tumors have been found to contain subpopulations of cancer cells that differ in their energy-generating pathways. One subpopulation consists of glucose-dependent (‘‘Warburg-effect’’) cells that secrete lactate, whereas cells of the second subpopulation preferentially import and utilize the lactate as their main energy source, functioning symbiotically. Soft tissue sarcomas are extremely heterogeneous since they arise in a multitude of tissues of many different cell lines, so this mechanism of energy symbiosis could be characteristic of their biological behavior. Glycolytic fueling can also activate oncogenes (RAS, MYC) and mutant tumor suppressors (TP53), which can be found in some syndromes associated with sarcomas (Carvalho et al. 2011).

The ^18^F-FDG is transported into the cells like glucose at a much higher rate, then it is phosphorylated to FDG-6-phosphate by hexokinase or glucokinase action, and does not enter the standard metabolic pathways, and can leave the cell only slowly by the action of glucose-6-phosphatase, so it is trapped in the neoplastic cells (Miele et al. 2008). Several hexokinase subtypes have also been described. Hexokinase I and in particular hexokinase II (HK-II) have been found to be expressed in tumors. Some authors suggest that HK-II regulating glucose metabolism in cancer cells, and the phosphorylation step may be rate-limiting in the FDG uptake and also suggest that the lack of glucose-6-phosphatase activity in tumors plays a role in determining FDG retention by preventing dephosphorylation (FDG-6-P to FDG). Thus, higher levels of GLUT do not guarantee increased ^18^F-FDG uptake by cancer cells, and metabolic trapping appears more likely as the rate-determining step in ^18^F-FDG uptake and maintaining the rapid uptake of glucose via GLUT-1 (Yang et al. 2019). Studies have shown that expression of GLUT1 and HK-II in epithelial cancer cells, including breast, esophageal, and lung cancer cells, plays a pivotal role in glucose metabolism and that the expression levels of GLUT1 and HK-II are correlated with malignancy (Tsukada et al. 2012). Sarcomas, like other tumors, display abnormal metabolic activity patterns, and detailed data regarding the STS metabolome is relatively sparse. Different oncogenes and tumor suppressors implicated in metabolic pathway regulation are mutated in sarcomas, like PIK3CA, and NF1. Furthermore, hypoxic tumor microenvironments, characteristic of sarcomas, modify metabolism and correlate with worse prognosis. Sarcoma cells display elevated glucose uptake and turnover, and glycolysis may be a key feature for tumor growth, correlated with his biological features (Esperança-Martins et al. 2021).

^18^F-FDG PET can be used to obtain quantitative parameters according to the tissues metabolic activity. There are different methods to quantify ^18^F-FDG activity, and compartmental models have been developed to calculate its concentration, to create a standardized method that allows comparable measurements. The standardized uptake value (SUV) is the most widely used quantitative imaging biomarker, also known as semi-quantitative analysis (Kinahan and Fletcher 2010; Thie 2004). Most STS have a high affinity for ^18^F-FDG, which is why PET/CT has been proposed as a non-invasive method, useful in diagnosis and follow-up. A significant relationship between the metabolic parameters of ^18^F-FDG PET/CT and the histological grade in STS has been found (Rakheja and Probst 2013; Ioannidis and Lau 2003; Macpherson et al. 2018). Volume-based metabolic parameters, such as metabolic tumor volume (MTV) and total lesion glycolysis (TLG), provide the total volume and total activity of metabolically active tumor cells (Hong et al. 2014). This work aims to establish the correlation between SUV (SUV_max_, SUV_mean_, and SUV_peak_), MTV, and TLG values of the baseline ^18^F-FDG PET/CT and the histological grade (FNCLCC) in patients with STS. Likewise, we analyze the usefulness of SUV, and volume-based metabolic parameters of baseline 18F-FDG PET/CT, as prognostic factors in overall survival (OS) and disease-free survival (DFS).

## Materials and methods

A retrospective study was conducted on patients diagnosed with STS who had histological grade according to the FNCLCC criteria, as well as a baseline ^18^F-FDG PET/CT (hereinafter PET/CT). The European Association of Nuclear Medicine (EANM) recommendations for the administered activity of ^18^F-FDG and study acquisition were followed (Boellaard et al. 2015). SUV_max_, SUV_mean_, SUV_peak_, and MTV values ​​were obtained for the main lesion. A VOI (volume of interest) was performed using the semi-automatic MVBT (maximum voxel-based thresholding) technique, with a threshold equal to 40% of SUV_max_ (Vanderhoek et al. 2013). For lesions with SUV_max_ less than 3, and those located near to physiological activity, an adaptive threshold-based spherical VOI was used, adjusted to a SUV_max_ threshold of 2 (Cheebsumon et al. 2011). The TLG was calculated according to the literature (Larson et al. 1999; Mucientes et al. 2018).

The quantitative variables were expressed as means and standard deviation, and the qualitative values as frequencies and percentages. The usefulness of metabolic biomarkers (SUV, MTV, and TLG values) as predictor variables of the histological grade was evaluated, using receiver operator characteristic (ROC) curves, calculating the area under the curve (AUC) with a 95% confidence interval (CI). A T-student test was performed to establish the relationship between the metabolic biomarkers measured in the baseline study and the histological grade. A survival function study was performed using the Kaplan–Meier method, calculating the OS and DFS. To assess the prognostic utility of the metabolic biomarkers, we use the Log-Rank method. The values of each variable measured in the baseline study were ordered, and two groups were created with the median value as the cut-off point. Paired OS and DFS curves were developed, looking for significant differences between them. All results were considered significant with alpha level < 0.05. The analysis was performed with SPSS v.20.0.

## Results

**Fig. 1:**
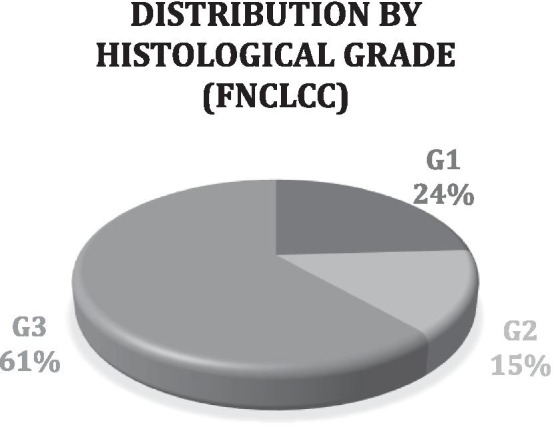
Distribution by histological grade (FNCLCC) in the baseline ^18^F-FDG PET/CT

**Fig. 2:**
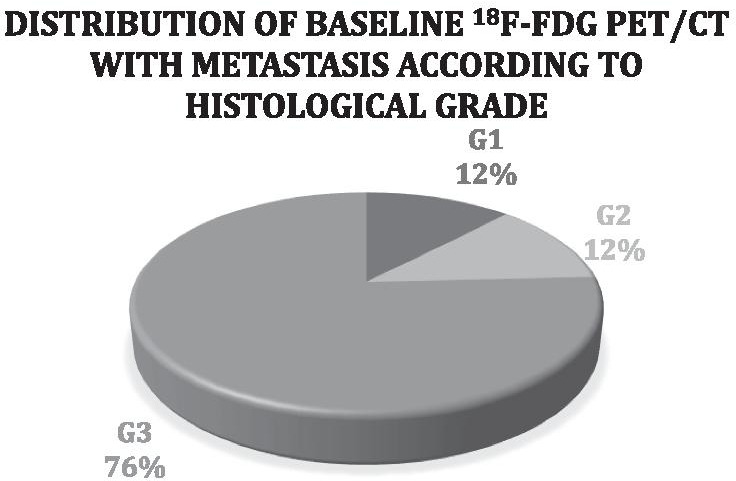
Distribution of baseline ^18^F-FDG PET/CT with metastasis according to histological grade

Eighty-three patients, 42 men, and 41 women were analyzed. The mean age at diagnosis was 50.62 years. Median blood glucose was 128 mg/dl (range 78–180 mg/dl). Seventy-six percent of the STS were high-grade (grades 2 and 3 according to the FNCLCC) (Fig. 1). Forty percent of the patients had metastases in the baseline PET/CT of which 87.88% were high-grade (Fig. 2, Table 1). The most common tumor types were adipocytic tumors (31%), smooth muscle tumors (16%), fibroblastic/myofibroblastic tumors (13%), and undifferentiated or unclassified sarcomas (13%). The most frequent tumor subtypes were liposarcomas (34%), leiomyosarcomas (14%), and undifferentiated pleomorphic sarcomas (12%). The most frequent locations were the lower limbs (45%), abdominal and retroperitoneal (30%), and the upper extremities (12%). The TLG and SUV ​​(SUV_max_, SUV_mean_, SUV_peak_) median values in the baseline PET/CT were higher in the high-grade than in the low-grade STS (Table 2).

**Table 1:** Metastases and locations in high-grade and low-grade tumors in the baseline ^18^F-FDG PET/CT

	Metastases and locations in high-grade and low-grade tumors					
	Lung (%)	Liver (%)	Surrounding soft tissue structures (%)	Bone (%)	Lymph nodes (%)	Intra-abdominal metastases (%)
LOW-GRADE	14.29	0	14.29	42.86	14.29	14.29
HIGH-GRADE	20.51	5.13	25.64	15.38	20.51	12.82

**Table 2:** Median values of the metabolic biomarkers measured in the baseline ^18^F-FDG PET/CT according to the histological grade

	Median values				
	SUV_max_	SUVmean	SUVpeak	VMT (cm^3^)	TGL
Low-grade	3.32	2.04	2.73	141.18	250.60
High-grade	8.80	3.99	7.17	86.83	336.03

**Fig. 3:**
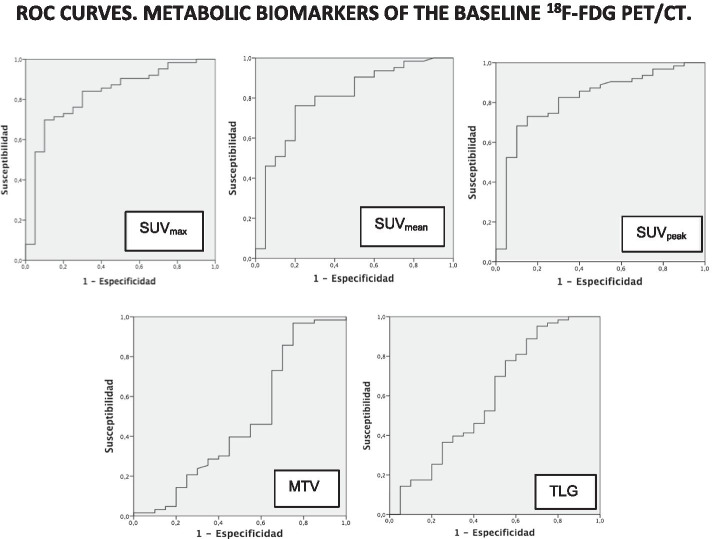
ROC curves. Metabolic biomarkers of the baseline ^18^F-FDG PET/CT

The usefulness of the metabolic parameters measured in the baseline PET/CT to discriminate high-grade from low-grade soft tissue sarcomas, was established using ROC curves, setting the following values as significant cut-off points: SUV_max_ 3.9 [AuC 0,824 (95% CI 0.717–0.931)], SUV_mean_ 2,5 [AuC 0.798 (95% CI 0.682–0.914)] and SUV_peak_ 3.73 [AuC 0.817 (95% CI 0.708–0.926)], being the SUV_max_ value the most accurate. The MTV and TLG parameters did not allow to define a significant cut-off point (Fig. 3). The T-student test found a statistically significant relationship between the histological grade and the metabolic parameters of the baseline PET/CT in the SUV_max_ [*p* = 0.04 (95% CI − 10.12 a − 1.93)], SUV_mean_ [*p* = 0.01 (95% CI − 3.62 a − 0.42)], and SUV_peak_ [*p* = 0.006 (95% CI − 8.35 a − 1.44)] values. No statistically significant relationship was found for the MTV (*p* = 0.36) or TLG (*p* = 0.34) values.

**Fig. 4:**
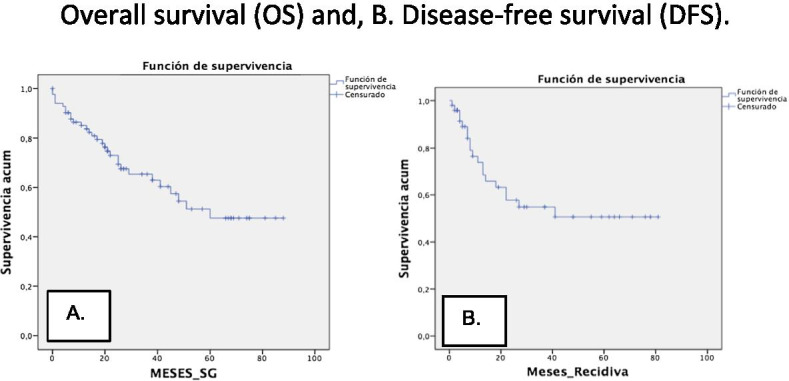
**A** Overall survival and **B** disease-free survival

**Fig. 5:**
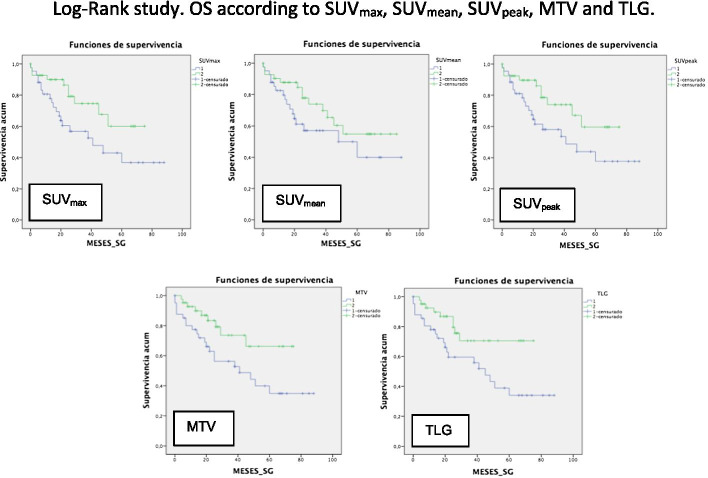
Log-Rank study. OS curves according to SUV_max_, SUV_mean_, SUV_peak_, MTV and TLG values in the baseline PET/CT

**Fig. 6:**
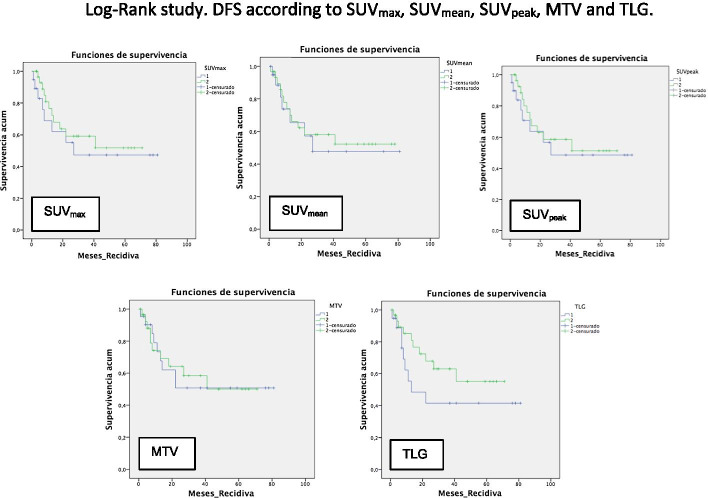
Log-Rank study. DFS curves according to SUV_max_, SUV_mean_, SUV_peak_, MTV and TLG values in the baseline PET/CT

A survival study was performed using the Kaplan–Meier method [OS 55.2 months (95% CI of 46.5–64)], [DFS 47.9 months (95% CI 36.9–58.8)] (Fig. 4). The Log-Rank study confirmed the prognostic value of baseline PET/CT SUV_max_ [*p* = 0.037 (95% CI 12.5–69.4)], SUV_peak_ [*p* = 0.05 (95% CI 13.1–68.8)], MTV [*p* = 0.022 (95% CI 7.8–74.1)], and TLG [*p* = 0.022 (95% CI 30.9–59)] values for OS. There were no significant differences in the OS for the SUV_mean_ (*p* = 0.141), or in the DFS for SUV_max_ (*p* = 0.51), SUV_mean_ (*p* = 0.74), SUV_peak_ (*p* = 0.60), MTV (*p* = 0.91), and TLG (*p* = 0.19) values (Figs. 5, 6).

## Discussion

The use of PET/CT in the initial staging and restaging of many cancers has been established (Rodríguez-Alfonso et al. 2014). Molecular imaging with PET/CT is certainly a powerful tool for the initial assessment of sarcomas and the detection of their recurrences (Charest et al. 2009 Dec). The ^18^F-FDG PET/CT is useful to differentiate malignant (SUV_max_ 0.6—14, mean: 5.9) from benign (SUV_max_ 1.2–6.2, mean: 3.5) lesions in STS patients (*p* < 0.001), with different uptake patterns according to the histological type (Nose et al. 2013). According to Massardo et al. (2012), the SUV_max_ value is related to histological grade, mitotic activity, and cellularity. Other authors have also found significant differences between the SUV_max_ values of high-grade and low-grade STS, regardless of the histological subtype (Bastiaannet et al. 2004). In a German study, the mean SUV_max_ value was 1.3 (range 0.37–1.9) for grade 1 STS, 2.7 (range 1.2–6) for grade 2, and 4.5 (range 1.4–9.1) for grade 3, with a statistically significant difference between them (*p* < 0.001) (Schwarzbach et al. 2000). For Benz et al. (Benz et al. 2010), a SUV_max_ value of 5.2 would be the optimal cut-off point (sensitivity: 74%, specificity: 91%, AbC: 0.85) to differentiate high-grade from low-grade STS. Our analysis also suggests that SUV values (SUV_max_ 3.9 [AuC 0.824 (95% CI 0.717–0.931)], SUV_mean_ 2,5 [AuC 0,798 (95% CI 0.682–0.914)] and SUV_peak_ 3.73 [AuC 0.817 (95% CI 0.708–0.926)], can be used to distinguish between high-grade and low-grade STS, being the SUV_max_ value the most accurate. Likewise, we found a significant relationship between the histological grade (FNCLCC) of the STS, and the SUV_max_, SUV_mean_, and SUV_peak_ values measured in the baseline PET/CT [SUV_max_ [*p* = 0.04 (95% CI − 10.12 a − 1.93)], SUV_mean_ [*p* = 0.01 (95% CI − 3.62 a − 0.42)], and SUV_peak_ [*p* = 0.006 (95% CI − 8.35 a − 1.44)]. According to the Memorial Sloan-Kettering Cancer Center and the European Organization for Research and Treatment of Cancer, the incidence of metastasis increases with tumor grade, and 90% of STS patients that develop lung metastases have high-grade lesions (Chao and Goldberg 2000; Billingsley et al. 1999); 75.8% of our population had high-grade STS (grade 2 and 3 FNCLCC), and 87.88% of the patients who developed distant metastases had a high-grade STS.

Other quantification parameters that evaluate the total volume of the tumor have been developed. Larson et al. (1999), introduced in 1999 the volume-based metabolic parameters MTV and TLG, as possible metabolic biomarkers that could be relevant in clinical practice (Mucientes et al. 2018). Choi et al. (2013) concluded that the TLG has a greater accuracy to discriminate high-grade from low-grade STS (AuC 0.802) than the SUV_max_ (AuC 0.726) and MTV (AuC 0.681) values. These findings are not consistent with our results, since according to our analysis, the MTV and TLG parameters did not allow us to define a significant cut-off point to discriminate high-grade from low-grade STS; neither did we find a statistically significant relationship for the MTV (p-0.36) or TLG (p-0.34) values with the histological grade, which can be explained by Schwarzbach et al. (2000). He found a significant relationship between the metabolic activity of the tumor and the histological grade, regardless of size, since the SUV value increases with the histological grade, and not with the size of the lesion. This discordance could also be due to the heterogeneity of demographics, and histological characteristics of the analyzed patients.

In our results MTV was larger in low-grade tumors than high grades, and SUV_mean_ values were similar in high- and low-grade tumors. This is probably because semiquantitative methods are impaired by tumor tissue heterogeneity, which makes it almost impossible to sample the contribution of each component of tumor to the metabolic activity in the VOI. Nevertheless, a semiquantitative measurement may be just about sufficient for clinical application (Lucignani 2009). Our purpose was to determine the clinical value of SUV values and volume-based metabolic parameters of ^18^F-FDG Positron emission tomography/Computed tomography (PET/CT), using the semi-automatic techniques, since they are generally accepted quantification methods used in daily practice, easy to perform, and their correlation with more complex measurements and methods is usually good. Consequently, no data was tabulated or qualified regarding the visual characteristics of the lesions (e.g., heterogeneous uptake, necrosis). However, intratumoral metabolic heterogeneity and the presence of necrosis and the volume of necrosis, are adverse prognostic factors for disease recurrence and death in patients with STS (Rakheja et al. 2013, Son et al. 2014).

**Fig. 7.**
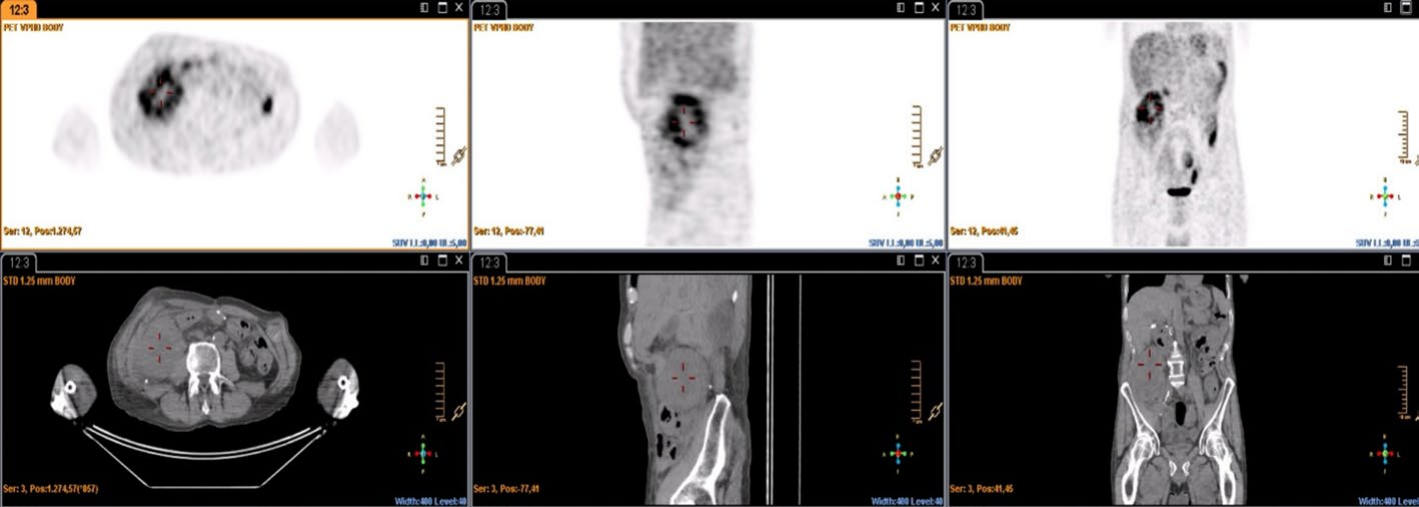
64-year-old male with high-grade spindle cell sarcoma. PET CT showed a mesenteric mass with heterogeneous ^18^F-FDG uptake

**Fig. 8.**
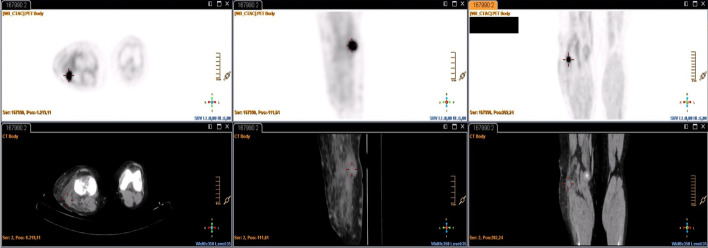
43-year-old female with low-grade malignant peripheral nerve sheath Tumor. PET CT showed a solid nodule in the right lower limb with homogeneous uptake of ^18^F-FDG

The SUV_max_, MTV and TLG values of the baseline PET/CT have prognostic value for OS, progression-free survival (PFS), and DFS. Patients with high SUV_max_, VMT, and TGL values have a lower survival (Li et al. 2016; Chen et al. 2017; Kubo et al. 2016). A meta-analysis concluded that SUV_max_, MTV and TLG of baseline PET/CT have prognostic value for OS in STS patients, being useful in identifying high-risk patients (Chen et al. 2017). Schuetze et al. (2005), found significant differences in DFS (*p* = 0.004) and in OS (*p* = 0.05), in patients who had a SUV_max_ value of less than 6 in the baseline study. Better OS and local recurrence have been found in patients with SUV_max_ less than 10.3 (*p* = 0.005 and 0.046, respectively) (Sambri et al. 2019 Jun). According to Hong et al. (Hong et al. 2014), SUV_max_ (*p* = 0.008) and SUV_mean_ (*p* = 0.032) are independent predictors of OS in STS patients. In our analysis, the mean OS was 55.2 months, and the mean DFS was 47.9 months. The log rank test outcomes suggest that the SUV_max_, SUV_peak_, MTV and TLG values of the baseline PET/CT were predictive variables of OS [SUV_max_ (*p* = 0.037), SUV_peak_ (*p* = 0.05), MTV (*p* = 0.022), TLG (*p* = 0.022)]. SUV_max_ values above 7.10, SUV_peak_ greater than 6.15, MTV greater than 102.52 cm3, and TLG greater than 335.07, were related to a lower OS. SUV_mean_ value did not correlate with survival but others, and it is probably a consequence of the similarity of the values of the SUV_mean_ in high- and low-grade tumors, due to tumor tissue heterogeneity in high-grade STS (Figs. 7, 8). This aspect of malignancy has long been known and has been described with the histologic features of cellular proliferation, necrosis, noncellular accumulations (matrix material and fibrous tissue), and physiologic characteristics (differences in blood flow, cellular metabolism, oxygenation, and expression of specific receptors). That heterogeneity in tumor metabolism recorded by ^18^F-FDG uptake is reflective of tumor biologic (Benz et al. 2008; Eary et al. 2008). Another limitation of SUV values normalized by total body weight is that they are affected by the amount of body fat. The SUV of normal tissues and lesions is higher (overestimated) in obese patients than in patients with a normal body mass index (BMI). SUL (SUV normalized by lean body mass [LBM]) is recommended for more accurate results. However, currently, SUL can be used only in obese patients, because the current standard values for differentiating benign from malignant lesions, and reference SUVs (liver, blood pool, and other tissues) have been calculated from patients with a normal BMI or from mixtures of patients with various BMIs. If SUL is going to be used in routine practice, the standard values should be determined in large numbers of people with standard BMI (Sarikaya et al. 2019a).

A high level of blood glucose competes with ^18^F-FDG and reduces its uptake in pathologic tissues, and endogenous insulin level also increases, which causes higher ^18^F-FDG uptake in insulin-sensitive normal tissues (fat and muscle). As a result, hyperglycemia can cause suboptimal differentiation of malignant from benign lesions, and underestimation of tumor grade (Sarikaya et al. 2019b). In our population, median blood glucose (BG) was 128 mg/dl (range 78–186 mg/dl), and patients with high blood glucose levels (over 200 mg/dl) were rescheduled or excluded, depending on the patient’s circumstances. Although guidelines recommend injecting FDG when blood glucose below 200 mg/dl, starting from BG > 110 mg/dl brain FDG uptake gradually and significantly reduces (Sarikaya et al. 2019b; Sprinz et al. 2018). Because there may be a similarity between brain and tumor glucose kinetics, and both brain and tumor show high, GLUT1 and GLUT3 expression (Sarikaya et al. 2019b), maybe tumor uptake is lower in patients with high glucose levels, and when comparing SUVs in high- and low-grade tumors, cases with blood glucose < 150 mg/dl at the time of FDG injection, and chronic hyperglycemia cases, should be avoided for better results for investigation purposes.

Our analysis suggests that SUV_max,_ SUV_mean,_ and SUV_peak_ values of baseline ^18^F-FDG PET/CT are useful to discriminate high-grade from low-grade STS, with a statistically significant relationship between histological grade and SUV values, being the SUV_max_ value the most significant. Furthermore, we found that patients with high SUV_max_, SUV_peak_, MTV, and TLG values have significantly lower OS. We can conclude that the ^18^F-FDG PET/CT is a useful tool in staging patients with STS, and its future incorporation in prognostic nomograms and clinical practice guidelines should be considered.

We consider that the limitations of our study are its retrospective nature, and the heterogeneity of demographics, and histological characteristics. Likewise, it was not possible to carry out an analysis by histological subtypes due to the limitation of the sample because of the low incidence of STS.

## Data Availability

The datasets used and/or analyzed during the current study are available from the corresponding author on reasonable request.
